# Impact of Physical Activity Practice and Adherence to the Mediterranean Diet in Relation to Multiple Intelligences among University Students

**DOI:** 10.3390/nu12092630

**Published:** 2020-08-28

**Authors:** José Luis Ubago-Jiménez, Félix Zurita-Ortega, Silvia San Román-Mata, Pilar Puertas-Molero, Gabriel González-Valero

**Affiliations:** 1 Department of Musical, Plastic and Corporal Expression, University of Granada, 18071 Granada, Spain; jlubago@ugr.es (J.L.U.-J.); felixzo@ugr.es (F.Z.-O.); pilarpuertas@correo.ugr.es (P.P.-M.); ggvalero@ugr.es (G.G.-V.); 2 Department of Nursing, University of Granada, 18071 Granada, Spain

**Keywords:** multiple intelligence, Mediterranean diet, physical activity, university students

## Abstract

Physical activity is important at any time of life. Particularly in the university, people tend to have more sedentary life, due to their studies. Eating habits are another health factor to consider. In addition, the Multiple Intelligences theory is a proposal that seeks the integral development and well-being of people. A descriptive, cross-sectional, and non-experimental research with the purpose of this study is to establish the relationships between practice of physical activity and the intelligences and determine the relationship between diet and the different types of intelligence in 215 university students. Findings indicate higher adherence to Mediterranean Diet in women and higher physical activity scores in men. Regarding multiple intelligences, men have higher indices in Bodily-kinesthetic, Interpersonal, Logical-mathematical, Musical, and Spatial intelligences, while women show higher levels in relation to Linguistic, Intrapersonal, and Naturalistic intelligences. Main conclusions from this study suggest the relationship between multiple intelligence and healthy habits, while also highlighting the need to improve eating habits and achieve greater adherence to Mediterranean Diet.

## 1. Introduction

The university stage is replete of numerous changes at the personal, social, and academic levels [1,2]. Likewise, all people have different skills and abilities. The concept about development of intelligence/skills as essential to accompany human beings throughout their lives has changed. Therefore, intelligences are understood as biological abilities, requiring the development of specific brain areas, which rarely work themselves [3,4]. This new tendency makes it possible to overcome the paradigm that placed learning based on exclusively genetic components, with the intelligence quotient (IQ) as the principal indicator. Consequently, it is necessary to attend personal and developmental needs in a customized form, because each individual to a greater or lesser extent developed type of intelligence [5,6]. Therefore, recognizing the student according to their capacities and strengths becomes an essential need for teaching at all educational levels.

The multiple intelligences (MI) theory shows how all people have a specific skill and a type of intelligence to a greater or lesser extent [7]. However, there are eight different intelligences with a specific location in the brain [8]. Focusing on the eight examples of knowledge, they are defined as:

− Naturalistic: The skill to recognize the order in nature and to organize and categorize the natural world.
− Bodily–kinesthetic: Ability to express and solve problems through the body.
− Linguistic: Ability to use words and language effectively, and sensitivity to writing and communication.
− Logical–mathematical: High aptitudes for the use of numbers in an effective way, to recognize abstract patterns, to decipher relations, and of reasoning.
− Spatial: Ability to visualize and remember objects and spatial dimensions and to recreate mental images and illustrations.
− Musical: Ability to recognize tonal patterns and sounds, as well as sensitivity to rhythms.
− Interpersonal: Ability to understand people’s moods, feelings, motivations, and intentions, as well as the leadership of groups of people.
− Intrapersonal: Ability to know oneself, reflect, and practice self-discipline.

This theory explains how each intelligence is developed in those activities which belong to them. Intelligence acquisition depends on cultural and environmental factors. Likewise, these abilities are related to individuals’ experience and knowledge. Therefore, it can be trained [5,9].

Likewise, in the university stage, many eating habits are set aside, and ultra-processed products are consumed more frequently [10,11]. This factor of change makes it necessary to have a varied diet in foods such as the Mediterranean. It provides an improvement in living habits and provides the necessary nutrients [12,13]. The Mediterranean diet (MD) is rich in vegetables, fruits, legumes, nuts, and cereals, with olive oil as the basic dietary fat [14]. In this regard, MD is high in antioxidants, fibers, and polyunsaturated fats that reduce risk of different types of cancer [15]. The particularities of the MD not only include a wide variety of foods, but also a certain amount and consumption of these foods is recommended. However, particular emphasis is placed on the importance of food preparation [16,17]. In recent years, the proliferation of research pointing to and detecting the benefits of the nutritional components inherent in the MD on the contribution to health benefits, which are related to an increase in life expectancy, a decrease in the risk of suffering from cardiovascular disease, diabetes, infections, or cancer [18,19].

Moreover, maintaining a good diet such as MD has positive effects on brain development and thus on intelligence [20,21]. In parallel, MD has been studied for its multiple benefits against cognitive impairment [22,23]. Numerous studies point out how MD foods, rich in omega-3s, support brain development [24,25]. Another factor that has a direct impact on the development of intelligence is the practice of physical activity (PA) [26]. When people practice sport, their organism produces a variety of neurotransmitters such as dopamine, serotonin, or norepinephrine which, among other factors, improve cognitive performance [27,28]. In addition, university students are often required to spend long periods of time in a sedentary environment in order to cope with their studies [29]. Consequently, it is necessary to develop adherence to physical activity to assist them in concentration [30], intellectual development [31,32], and cardiovascular health improvement [33].

Recent research suggests that different types of exercise promote cognitive function through neurobiology [34,35,36]. Aerobic training and resistance training impact the brain. Both influence the activation of the functional cortex [37] and increase the volume of the hippocampus [30]. The potential of this research is to unify the factors described, i.e., MI, MD, and PA, with the intention of examining the relationships between them and providing a university student profile by gender, based on the scores obtained on the instruments administered. Specifically, according to previous studies, within the theory defined by Gardner, special emphasis will be placed on bodily–kinesthetic intelligence, as it is intimately related to physical activity [38].

Accordingly, three hypotheses emerged in the present study. The first one assumes that “there are differences between MD, MI, and PA according to gender in favor to males”. Secondly, we consider that there is a relationship between diet and MI. Specifically, we argue that “education students who have an optimal diet tend to have higher levels of bodily–kinesthetic intelligence”. We also consider that there exists a relationship between PA and MI. In this regard, the third hypothesis of this study is “students who practice PA more often tend to have higher levels of MI”.

Taking into account the research questions defined above, the present research was designed with the following general aims: (a) Assess the intensity of individual dimensions of MI and the degree of implementation of the MD and the level of PA in undergraduates; and (b) analyze the relationship between MD, PA, and MI depending on gender.

## 2. Materials and Methods

### 2.1. Subjects and Design

This descriptive, cross-sectional, and non-experimental research project was carried out using a sample of 215 students who attended between the first and fourth year of their degree in the University of Granada (Granada, Spain), with a homogeneous distribution according to sex, representing 59.1% (*n* = 127) females and 40.9% (*n* = 88) males. Convenience sampling was used to recruit the participants. The age of the participants was between 18 and 48 years old (21.12 ± 4.34). It should be noted that 15 questionnaires were invalidated because they were not properly completed.

### 2.2. Instruments

Three different types of instrument were used in this research project. The first was an ad hoc questionnaire where undergraduates indicated their sex, their age, and academic year they were in.

In addition, in order to measure the level of physical activity exercised, questions were included in the ad hoc questionnaire regarding the time in minutes of moderate or intense physical activity performed each week and their relative personal capacity. Participants answered according to a Likert scale ranging from 0 (no physical activity or sport) to 10 (above their capacity). Core scores of 5 or 6 equaled moderate physical activity. Once these data were collected, the recommendations for physical activity made by the World Health Organization were taken into account [20]. Thus, the categories were whether people met minimum or active levels (HPA > 150 min of moderate physical activity) and did not meet minimum or sedentary levels (SPA < 150 min of moderate physical activity). The physically active category was defined as ≥150 min per week of physical activity.

Adherence to the MD was assessed using the Mediterranean Diet Quality Index for children and adolescents (KIDMED) questionnaire [39], which contains 16 questions to be answered in a positive or negative way (yes/no). Affirmative answers in questions representing a negative connotation in relation to the Mediterranean diet are worth −1 point and positive answers in questions representing a positive connotation in relation to the Mediterranean diet are worth +1 point. Negative answers do not score. To represent what type of adherence each subject showed, values were assigned to each term as follows: Values between 8 and 12 (both included) were defined as an optimal diet, values between 4 and 7 were understood as an improbable diet, and values between −4 and 3 were understood as a low-quality diet.

The Multiple Intelligence Survey (MIS) [40] was used. The questionnaire is formed by 3 blocks of 9 items measuring each of the 8 intelligences: Linguistic (L); Logical-mathematical (LM); Bodily-kinesthetic (BK); Musical (M); Naturalistic (N); Spatial (S); Interpersonal (INTER); Intrapersonal (INTRA). Possible scores range from 3 to 27 with a midpoint of 15. Participants have to rank all items starting with 1 for the item the most like them to 9 for the item least like them.

### 2.3. Procedure

Firstly, the Faculty of Education of the University of Granada (Granada, Spain) gave permission to researchers (1478/CEIH/2020). Afterwards, a document was drafted explaining the study nature and aims and requesting the consent of students who would like to participate. Once an affirmative response was received, the questionnaire was sent by email so that participants could respond honestly. A total of 230 students took part in this project, and 15 questionnaires were invalidated because they were not correctly completed. The instruments were applied from February to March 2019. Anonymity and confidentiality of the data were ensured. Data were collected and their quality was confirmed, whilst ensuring throughout that the process conformed to the ethical principles for research defined in the Helsinki’s Declaration in 1975 and subsequently updated in Brazil in 2013.

### 2.4. Statistical Analysis

For data analysis, the statistical software IBM SPSS 25.0. (International Business Machines Corporation, Armonk, NY, USA) was used in order to establish the values of the basic descriptors (means and frequencies). The magnitude of the differences or effect size was determined using Cohen’s standardized measure d, and was interpreted as zero (0–0.19), low (0.20–0.49), moderate (0.50–0.79), or high (≥0.80). Therefore, for each effect size, the 95% confidence interval (95% CI) was calculated. In order to understand differences between two correlations, the effect size was calculated with Cohen’s q. Both correlations were converted to Fisher’s Z and subtracted from each other. This measure can be interpreted as: ≤0.1 (no effect), 0.1–0.3 (small effect), 0.3–0.5 (medium effect), and ≥0.5 (large effect). On the other hand, the differences between categorical variables were determined through contingency tables. Likewise, the differences between variables of categorical and interval type were analyzed using ANOVA and a Student’s *t*-test. Likewise, the Bonferroni test was applied to verify inter-group differences. We also performed linear regression analysis to study the association between adherence to MD and PA (independent variables) and MI (dependent variable), adjusted for gender.

## 3. Results

The results achieved in the present investigation variables are shown in Table 1 in relation to participants’ gender. A statistically significant relation was found in four of the eight intelligence categories, as well as in adherence to the MD and PA practice (*p* < 0.05). For BK intelligence, women showed higher average values (17.16 ± 6.23) than men (16.01 ± 7.06) (*p* = 0.022). There were also higher average values for females in the INTER, M, and S intelligences (16.46 ± 5.62; 17.17 ± 5.76; 16.06 ± 5.33), respectively (*p* = 0.028; *p* = 0.020; *p* = 0.031), than males (15.80 ± 7.06; 15.35 ± 5.20; 14.83 ± 4.95), respectively. At a significant level of *p* < 0.001, it was noted that women have higher average levels in KIDMED than men (8.86 ± 2.46 vs. 6.75 ± 2.48). However, males have higher average in PA (4.07 ± 0.52) than females (3.86 ± 0.49). In addition, there were also statistically significant differences *p* = 0.030 between women and men who perform HPA. In contrast, there were also statistically significant differences in terms of SPA (*p* < 0.001), where men have a higher average than women.

Table 2 shows the correlations between the different investigation variables in relation to the female. The strongest relationships were found among women who have developed LM and have greater skills in INTRA intelligence (*r* = 0.748), with BK intelligence (*r* = 0.669), with S intelligence (*r* = 0.616), with M intelligence (*r* = 0.603), and with N intelligence (*r* = 0.585). On the other hand, the BK intelligence presented a strong correlation with the INTRA (*r* = 0.670), INTER (*r* = 0.633), S (*r* = 0.573), M (*r* = 0.563), and N (*r* = 0.539) intelligences. Likewise, for DM only correlations with INTER intelligence (*r* = 0.630) were found. Finally, for PA no correlation was found with any type of intelligence although a negative correlation was obtained with MD (*r* = −0.233).

Finally, correlation coefficients were shown between multiple intelligences, MD adherence, and PA for males (Table 3). In relation to MD, the most significant result was the positive correlation with BK (*r* = 0.245) and N (*r* = 0.215), as well as establishing positive correlations with PA (*r* = 0.179). Based on PA, positive correlations were found with intelligence INTRA (*r* = 0.213). Among the different intelligences the strongest correlations were between the intelligence BK and INTER (*r* = 0.639), the M and the N (*r* = 0.638), between the INTRA with the LM (*r* = 0.629), BK (*r* = 0.617), and the M (*r* = 0.602). Likewise, within the associations among the different intelligences, M shows a positive correlation with S (*r* = 0.600); LM correlates positively with BK (*r* = 0.590), M (*r* = 0.580), and S (*r* = 0.554). Likewise, for MD, correlations were found with the BK (*r* = 0.245) and N (*r* = 0.215) intelligences. Correlations were found for PA with the INTRA intelligence (*r* = 0.213) and with MD (*r* = 0.179).

Linear regression analyses were performed to check the association between PA and MD for each of the eight intelligences. The regression was also carried out differentiating between men and women (Table 4). For the predictive models in relation to the male sex, only PA was a predictive variable of intelligence BK (*β* = 0.157; *p* = 0.015), which explains 39% of the variance of the response variable. On the other hand, in the case of women, MD was a predictive variable of INTER intelligence (*β* = 0.326; *p* = 0.025), explaining 11% of the variance. For the rest of the intelligences none of the variables introduced were significant predictors.

## 4. Discussion

Maintaining a healthy lifestyle is associated with doing PA and adopting a healthy diet. Moreover, considering young people are involved in a life process requiring several changes in personal, physical, and psychological spheres, the main objective of this research was to identify the relationships between eating habits, PA practice, and MI in undergraduates. In addition, intelligence is also influenced when acquiring those habits. It also intends to establish conclusions to help understand the relationship between the different variables. Therefore, it is necessary to promote healthy habits [12,17,41,42]. Some studies of similar characteristics [43,44,45] have been conducted among undergraduates. However, no studies have been conducted associating healthy habits and MI.

In the first hypothesis of the present research, differences between variables considered according to gender existed. This hypothesis was partially confirmed. While we considered that men would score higher on all three variables, the talk obtained showed that men obtained higher PA scores than women. As for MI, similar scores were observed in relation to gender, although the highest score was obtained by women in M intelligence. Concerning MD, women obtained higher scores. Accordingly, findings suggested that undergraduates with moderate to high adherence to MD do regular exercise. Likewise, it showed how females, even though their diet levels are higher, had less PA practice than males. Similar findings can be observed in the study by Chacón-Cuberos et al. [46] showing how women have a better diet in relation to men. The current research shows how women adhere better to MD even though they have lower PA than men [47,48]. In contrast, similar studies [49,50] found a positive relationship between adherence to MD and PA with no differences observed between men and women.

In relation to the second hypothesis considering a relationship between having high levels of MD and BK intelligence, the hypothesis was confirmed in the case of men, along with the N one. For women, a relationship with INTER intelligence was found. Although there are no studies that connect adherence to MD with MI, there are studies that identify nutritional habits as contributing to better brain functioning and thus intelligence [51]. In addition, several studies have found significant positive effects on health from the ingestion of varied foods such as vegetables, fruits, fish, meat, or olive oil that constitute MD [52,53,54,55]. Findings obtained disagree with the study by Jackson and Beaver [51] who establish a relationship between healthy eating habits and verbal intelligence. MD is also positively linked to verbal intelligence in other studies [52].

For the analysis of third hypothesis of the present research, it was important to focus on the levels found for MI. Thus, the results shown in relation to multiple intelligences show how the findings were similar between men and women. It is important to highlight how male undergraduates showed higher levels in relation to BK, INTER, LM, M, and S intelligences. In contrast, females showed higher levels in relation to L, INTRA, and N. These results are similar to findings being obtained by Oh et al. [49] but they were different from the results obtained by Furnham and Shagabutdinova [50] and Chan [56]. These investigations show how women showed higher levels in INTRA, INTER, M, and L than men. These values are linked to PA, because BK intelligence and sports practice were higher in men. Moreover, it has been confirmed by several empirical researches [57,58,59]. However, those findings differ from the findings reported by Ermis and Imamoglu [57], which analyzed how exercise affects multiple intelligences. It was found in this study involving 1580 students that women scored higher levels in visual and musical intelligence and lower in intrapersonal intelligence than men.

Regarding the intensity of PA, it was found that men present higher values than women in relation to HPA. Similar data are found in several studies carried out on university students [60,61,62]. Likewise, men obtained higher results than women in relation to SPA. These findings differ from the review by Panadero–Pérez et al. [63] among adolescents and young adults. It should also be noted that these findings offer a starting point for research into the impact of PA practice and maintaining a diverse diet full of food items such as MD. A further research direction is the establishment of an intervention based on the practice of PA, a quasi-experimental study, to measure the relationship and the more detailed influence between diet and PA on the development of intelligences among students.

The present research also had some limitations. Firstly, it was carried out with undergraduates to ensure the sample was compared with similar studies. In addition, the study employed a descriptive and cross-sectional design providing useful information on this emerging topic but avoiding casual conclusions. Another limitation refers to the variables used, since although these are valid for fulfilling the study’s aims, it would have been interesting to include others of great relevance such as the body mass index or total daily energy intake. The MIS questionnaire is also another limitation, since it evaluates a person’s self-knowledge of their own tastes, so it would be interesting to introduce a questionnaire that measures intelligence objectively. This would make it possible to further investigations the relationship between MI, eating habits, and health status. However, the strength of the correlations was generally low, even though significant relationships were developed for MD, PA, and MI. Recognizing the above limitations, we make the following suggestions for future research. This study should be replicated and expanded with other samples and exploring other variables, such as psychosocial factors or emotional intelligence. In addition, it would be interesting to develop an intervention program to check out the combined influence of MD and PA on the development of MI and the academic performance of undergraduates.

## 5. Conclusions

The main conclusions of this research show the importance of improving dietary habits and achieving greater adherence to MD, with a special emphasis on consumption of fruits, vegetables, fish, cereals, and nuts. In addition, various intelligences were positively associated with these healthy habits. Based on the initial hypotheses, it was corroborated that women undergraduates show better eating habits and men show a higher rate of physical activity. It is necessary to promote healthy habits, as physical and cognitive benefits are achieved in time. Adhering to a specific program of MD and regularly exercise could improve different intelligences of further professionals. These findings support the need to promote physical activity and provide basic nutritional education to improve people’s quality of life. Furthermore, the relationships found between some of the intelligences included in Gardner’s theory point to an important way to achieve this. In fact, the main practical implications of the present study are oriented to the consolidation of evidences for the design and development of healthy programs in higher education, which promote healthy lifestyles among young university students. Further research will be oriented to the development of longitudinal research, with larger samples where the relationship between the variables considered can be examined in more detail and the real impact of healthy education based on the theory of multiple intelligences for the university population can be known.

## Figures and Tables

**Table 1:** Multiple intelligence, Mediterranean diet, and physical activity practice according to the participants’ gender.

Variable	Male		Female		Levene Test		Sig. (Bilateral)	ES (d)	95% CI
	M	S.D.	M	S.D.	F	Sig.			
**BK**	16.01	7.06	17.16	6.23	6.29	0.01	0.022 *	0.161	(−0.111; 0.433)
**INTER**	15.80	5.99	16.46	5.62	16.74	0.00	0.028 *	0.114	(−0.158; 0.386)
**INTRA**	17.69	6.17	16.92	6.63	2.57	0.11	0.173	0.119	(−0.153; 0.392)
**LM**	15.26	5.76	16.88	5.79	3.03	0.08	0.921	0.281	(0.007; 0.553)
**M**	15.35	5.20	17.17	5.76	0.17	0.67	0.020 *	0.328	(0.055; 0.602)
**N**	16.89	6.06	15.90	6.47	3.90	0.04	0.544	0.157	(−0.115; 0.429)
**L**	14.49	5.54	14.05	5.89	0.01	0.89	0.754	0.076	(−0.196; 0.348)
**S**	14.83	4.95	16.06	5.33	0.39	0.53	0.031 *	0.237	(−0.035; 0.510)
**KIDMED**	6.75	2.48	8.86	2.46	6.39	0.00	0.000 *	0.853	(0.853; 1.136)
**PA**	4.07	0.52	3.86	0.49	3.17	0.65	0.000 *	0.413	(0.139; 0.688)
**HPA**	3.92	1.02	3.24	0.81	0.04	0.82	0.030 *	0.312	(0.141; 0.424)
**SPA**	2.46	0.67	2.20	0.74	0.01	0.91	0.000 *	0.230	(0.241; 0.474)

Note: Effect size Cohen’s d (ES); 95% Confidence Interval (CI); Bodily-kinesthetic (BK); Interpersonal (INTER); Intrapersonal (INTRA); Logical-mathematical (LM); Musical (M); Naturalistic (N); Linguistic (L); Spatial (S); Mediterranean Diet Adherence (KIDMED); Physical Activity (PA); Physical Activity Healthy (PAH); Physical Activity Sedentary (PAS). * *p* < 0.05

**Table 2:** Bivariate correlations between multiple intelligences, PA, and MD for females.

	BK	INTER	INTRA	LM	M	N	L	S	MD	PA
**BK**		0.633 **	0.670 **	0.669 **	0.563 **	0.539 **	0.375 **	0.573 **	0.033	−0.142
**INTER**			0.508 **	0.519 **	0.448 **	0.381 **	0.311 **	0.380 **	0.630 **	−0.171
**INTRA**				0.748 **	0.665 **	0.649 **	0.396 **	0.670 **	0.008	0.002
**LM**					0.603 **	0.585 **	0.491 **	0.616 **	0.020	−0.068
**M**						0.539 **	0.436 **	0.573 **	0.016	0.078
**N**							0.427 **	0.454 **	−0.055	−0.037
**L**								0.423 **	0.116	−0.007
**S**									0.083	0.012
**MD**										−0.233 **
**PA**										

**: Correlation significant at 0.01 level (bilateral); *: Correlation significant at 0.05 level (bilateral); Physical Activity (PA); Bodily-kinesthetic (BK); Interpersonal (INTER); Intrapersonal (INTRA); Logical-mathematical (LM); Musical (M); Naturalistic (N); Linguistic (L); Spatial (S); Mediterranean Diet Adherence (MD).

**Table 3:** Bivariate correlations between multiple intelligences, PA, and MD for males.

	BK	INTER	INTRA	LM	M	N	L	S	MD	PA
**BK**		0.639 **	0.617 **	0.590 **	0.567 **	0.541 **	0.211 *	0.464 **	0.245 *	−0.005
**INTER**			0.516 **	0.415 **	0.566 **	0.504 **	0.120	0.469 **	0.124	−0.102
**INTRA**				0.629 **	0.602 **	0.578 **	0.315 **	0.542 **	0.144	0.213 *
**LM**					0.580 **	0.419 **	0.356 **	0.554 **	0.166	0.114
**M**						0.638 **	0.415 **	0.600 **	0.063	0.027
**N**							0.495 **	0.479 **	0.215 *	−0.047
**L**								0.466 **	0.182	−0.042
**S**									0.049	0.156
**MD**										0.179 *
**PA**										

**: Correlation significant at 0.01 level (bilateral); *: Correlation significant at 0.05 level (bilateral); Physical Activity (PA); Bodily-kinesthetic (BK); Interpersonal (INTER); Intrapersonal (INTRA); Logical-mathematical (LM); Musical (M); Naturalistic (N); Linguistic (L); Spatial (S); Mediterranean Diet Adherence (MD).

**Table 4:** Regression model for multiple intelligences, PA, and MD.

Variables	Standardized *β*		*t*		*p*		95% CI		R^2^		Adjusted R^2^	
	Male	Female	Male	Female	Male	Female	Male	Female	Male	Female	Male	Female
**Bodily-Kinesthetic** PA MD	0.157 0.134	0.144 0.430	−1.571 −0.343	−1.428 −0.341	0.015 0.118	0.098 0.215	(−4.05; 0.46) (−1.74; 1.68)	(−3.87; 0.35) (−1.55; 1.22)	0.402	0.114	0.397	0.094
**Interpersonal** PA MD	0.160 0.530	0.158 0.326	−1.772 0.594	−1.701 0.624	0.059 0.556	0.257 0.025	(−3.83; 0.21) (−1.08; 1.98)	(−3.64; 0.31) (−1.14; 1.33)	0.215	0.162	0.187	0.112
**Intrapersonal** PA MD	0.028 0.034	0.375 0.204	0.038 0.044	0.106 0.091	0.087 0.980	0.072 0.215	(−1.51; 1.58) (−1.97; 0.73)	(−1.28; 1.42) (−1.84; 0.85)	0.005	0.021	0.000	0.014
**Logical-Mathematical** PA MD	0.851 0.158	0.824 0.213	−0.789 −0.188	−0.841 −0.142	0.269 0.652	0.641 0.357	(−1.67; 0.59) (−1.87; 1.52)	(−1.74; 0.62) (−1.48; 1.05)	0.129	0.016	0.085	0.003
**Musical** PA MD	0.674 0.457	0.521 0.326	0.945 0.454	0.749 0.268	0.651 0.346	0.322 0.218	(−1.09; 3.11) (−1.23; 1.97)	(−1.12; 2.54) (−1.08; 1.67)	0.118	0.154	0.114	0.123
**Naturalistic** PA MD	0.840 0.544	0.754 0.564	0.595 0.922	0.678 0.523	0.063 0.358	0.478 0.235	(−2.87; 1.54) (−2.46; 0.89)	(−1.91; 1.23) (−2.32; 1.43)	0.184	0.218	0.175	0.137
**Linguistic** PA MD	0.264 0.115	0.185 0.174	0.186 1.263	0.286 0.727	0.853 0.209	0.677 0.258	(−1.82; 2.20) (−0.55; 2.51)	(−1.84; 2.01) (−0.84; 1.88)	0.113	0.167	0.097	0.094
**Spatial** PA MD	0.863 0.839	0.728 0.817	0.173 0.203	−0.254 −0.193	0.863 0.839	0.681 0.107	(−1.78; 2.12) (−1.33; 1.63)	(−1.66; 2.03) (−1.42; 1.52)	0.204	0.026	0.154	0.002

Note: *p* < 0.05; Physical Activity (PA); Mediterranean Diet Adherence (MD).
